# Systematic Sex-Based Inequity in the MELD Score-Based Allocation System for Liver Transplantation in Germany

**DOI:** 10.3389/ti.2025.13844

**Published:** 2025-01-29

**Authors:** Leke Wiering, Annette Aigner, Marieke van Rosmalen, Brigitta Globke, Tomasz Dziodzio, Nathanael Raschzok, Münevver Demir, Wenzel Schöning, Frank Tacke, Petra Reinke, Johann Pratschke, Robert Öllinger, Paul V. Ritschl

**Affiliations:** ^1^ Charité—Universitätsmedizin Berlin, Corporate Member of Freie Universität Berlin and Humboldt-Universität zu Berlin, Department of Hepatology and Gastroenterology, Campus Virchow-Klinikum and Charité Campus Mitte, Berlin, Germany; ^2^ Berlin Institute of Health at Charité—Universitätsmedizin Berlin, BIH Biomedical Innovation Academy, BIH Charité (Junior) Clinician Scientist Program, Berlin, Germany; ^3^ Charité—Universitätsmedizin Berlin, Corporate Member of Freie Universität Berlin and Humboldt-Universität zu Berlin, Institute of Biometry and Clinical Epidemiology, Berlin, Germany; ^4^ Eurotransplant International Foundation, Leiden, Netherlands; ^5^ Charité—Universitätsmedizin Berlin, Corporate Member of Freie Universität Berlin and Humboldt-Universität zu Berlin, Department of Surgery, Campus Charité Mitte, Campus Virchow-Klinikum, Berlin, Germany; ^6^ Charité—Universitätsmedizin Berlin, Corporate Member of Freie Universität Berlin and Humboldt-Universität zu Berlin, Berlin Center for Advanced Therapies (BeCAT), Berlin, Germany

**Keywords:** organ procurement and allocation, model for end-stage liver disease, end stage liver disease, disparities, gender, sex inequity, waiting list, access to transplantation

## Abstract

In liver allocation systems based on the Model for End-stage Liver Disease (MELD) score, sex inequities have been identified in countries with high organ donation rates. Whether similar inequities exist in regions with average to low donation rates remained unclear. We assessed the impact of sex on transplantation rates, waiting list mortality and post-transplant survival in 25,943 patients waitlisted for liver transplantation in Germany between 2003 and 2017 using competing risk analysis. Women are currently underrepresented on the waiting list (33.3%) and among transplant recipients (31.1%) compared to their proportion of severe liver disease cases (35.1%). The introduction of MELD-based allocation has worsened this disadvantage [HR before: 0.89 (0.81–0.98), after: 0.77 (0.74–0.81)]. Three key factors contribute to this disparity: Women have lower creatinine levels despite worse renal function, reducing their MELD score (median 1, 0–3). Second, exceptional MELD points are more frequently granted to men [HR 1.61 (1.54–1.69) compared to regular allocation]. Third, the small height of women has the highest impact on the probability of not being transplanted [adjusted HR 0.85 (0.81–0.9)]. Even in countries with lower organ donation rates, MELD-based allocation leads to sex inequity. Measures are needed to ensure sex-neutral liver allocation in MELD-based systems worldwide.

## Graphical Abstract

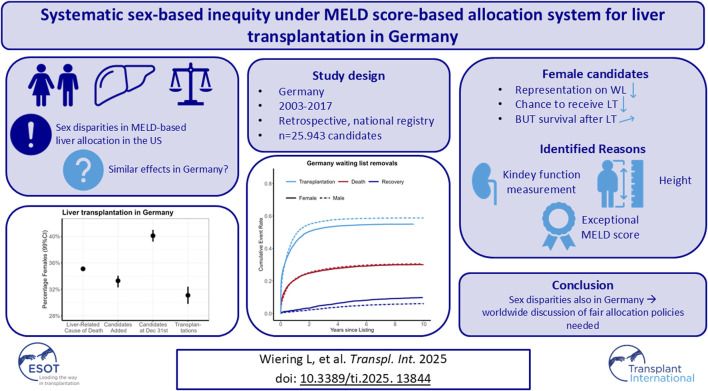

## Introduction

In recent years, sex disparities in liver transplantation have been increasingly recognized [1]. Among them, the chances of women to receive life-saving liver transplantation (LT) are reduced compared with those of men [2, 3]. Every year, more than 30.000 patients worldwide undergo LT [4] and the limited availability of deceased donor organs is still a problem of great ethical relevance that has not yet been solved.

In 2002, the United Network for Organ Sharing introduced the Model for End-stage Liver Disease (MELD) score as a new liver allocation policy in the United States [5, 6]. The MELD score is a disease-severity scale and aims to reduce waitlist mortality by using transparent criteria and guaranteeing fair allocation. The MELD formula counts total bilirubin (tBil), serum creatinine (sCr), and international normalized ratio (INR) [5]. Exceptions have been added for individuals whose disease severity is not adequately reflected by their actual calculated MELD score, assigning these patients exceptional MELD points [e.g., hepatocellular carcinoma (HCC), Supplementary Material S1, S2]. Today, the majority of countries offering LT have implemented comparable allocation rules [4]. In the United States, the liver allocation policy was recently changed to include sex (MELD 3.0) [7]. Although this represents an important advancement, the available data on sex disparities in liver transplantation are very much limited to the United States [8]. Although these data are crucial, they do not seem sufficient to adapt allocation policies worldwide as countries differ in their allocation procedures, access to healthcare, organ donation availability, and other factors. As a result, algorithms in other countries have not been adjusted for sex equity.

This study aimed to evaluate transplant probability in women in the context of the MELD-based liver allocation system in Germany, a country substantially different from the United States with respect to donation rates and access to healthcare, and to encourage possible amendments to overcome sex-based inequalities in liver transplantation worldwide.

## Materials and Methods

### Study Design and Setting

This study analyzed the German LT program. The primary endpoint was the hazard ratio for women compared with that for men to receive LT before and after the introduction of the MELD-based allocation system. The MELD-based allocation system was implemented on 16 December 2006. The study included patients registered (waitlisted) from 1 January 2003, to 31 December 2017.

### Data Sources and Quality

Data on the German LT program were provided by Eurotransplant with the approval of the working group for LT of the German Medical Association on 16 September 2019. Cause of death statistics were obtained from the Federal Statistical Office for Germany (Supplementary Material S4). Data were obtained in anonymized form.

### Patient Selection and Allocation Rules

To account for the applied allocation rules, pre-MELD and MELD eras were defined as the patients who were removed from the waiting list for any reason before or after the implementation of the MELD-based policy. Patients younger than 18, patients receiving a living donor transplant, patients receiving, or awaiting a multi-organ transplant and those listed with high urgency (equivalent to Status 1 in the United States) were excluded (see Supplementary Figures S1, S2).

### Variables and Definitions

Epidemiological and procedure-related data are listed in Supplementary Tables S1–S4. To address the bias resulting from the unisex use of sCr in the MELD formula despite physiologically lower sCr levels in women [9] their estimated glomerular filtration rates [eGFR using the chronic kidney disease epidemiology collaboration (CDK-EPI) formula [10]] were used. By inserting the female eGFR into the male formula for eGFR and back-calculating to sCr we determined a corrected sCr for women. Finally, a corrected MELD score was computed using this corrected sCr (Supplementary Material S3).

### Statistical Analysis

Descriptive statistics were carried out according to their level [absolute and relative frequencies for categorical variables, median and interquartile range (IQR) for continuous variables]. Cumulative incidence curves displaying time to transplantation, death or ineligibility (waiting list mortality), and recovery were plotted. To assess the effect of sex on transplantation, waiting list mortality and survival after transplantation we used competing risk analysis and derived cause-specific hazard ratios (HRs) based on multivariable Cox-proportional hazards regression models. Survival after transplantation was additionally depicted using Kaplan-Meier curves. Follow-up for survival analysis began at the time of transplantation and ended at death or was censored at the time of the last documented follow-up. The effect of height on transplantation was modeled using a spline with four degrees of freedom. We additionally ran sensitivity analyses with robust standard errors. Due to a very limited number of missing values in the key parameters of interest all models are based on complete cases (max. 0.04% for German data for all data presented in Supplementary Tables S1–S4). Statistical analyses were performed using R [11] (see Supplementary Material S5 for used packages). According to the local Institutional Review Board (Charité – Universitätsmedizin Berlin), no specific approval was required for this study, which analyzed data already anonymized.

## Results

### Sex Imbalance in the German Liver Transplantation System

In total, 25,943 patients were registered on the respective waiting lists during the observation period, of which 20,018 met the inclusion criteria. In fact, 10,482, (52.4%) of these patients underwent LT within the observation period. Patient demographics are summarized in Supplementary Figure S1; Supplementary Tables S1–S4 (candidates and recipients).

**FIGURE 1:**
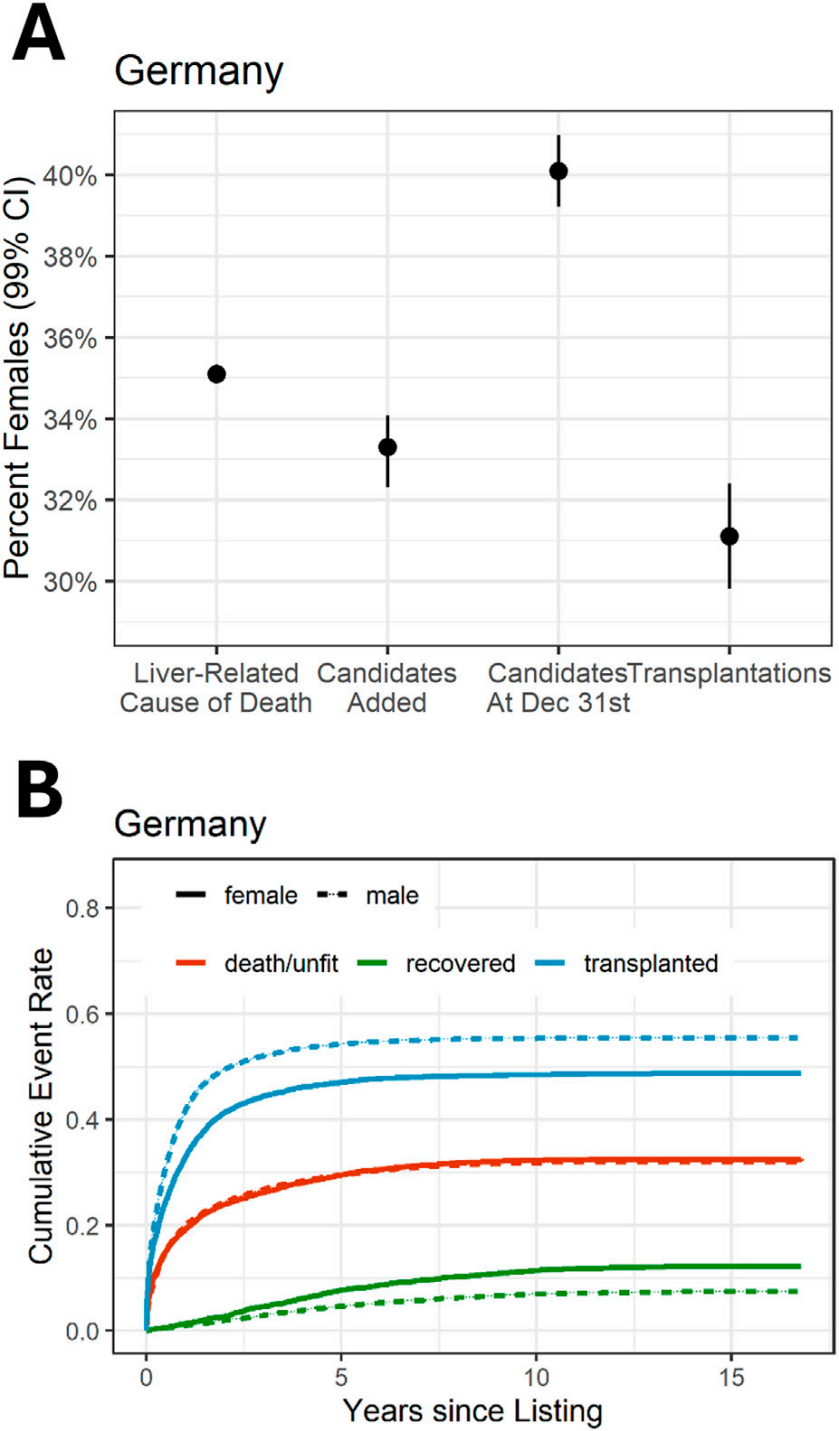
Sex ratio in liver transplantation and probability of transplantation by sex. **(A)** Female proportion in the German liver transplantation program in relation to liver-related causes of death. **(B)** Probability of outcome of candidates on the liver waiting list by time-to-event analysis (cumulative incidence function). The data displayed depict the MELD era for **(A, B)**.

Because the incidence of liver disease is not equally distributed between women and men, the proportion of liver-related causes of death was computed as a benchmark with women steadily representing 35.1%. The ratio of women newly registered on the waiting list and the percentage of waitlisted women who received LT considerably changed after the implementation of the MELD-based allocation system (Supplementary Figure S2). Annual waitlist registrations for women decreased, i.e., from 36.2% (95% confidence interval (95%CI): 34.5–37.9) to 33.3% (95%CI: 32.3–34.1), and the annual percentage of actual female transplant recipients decreased, i.e., from 34.4% (95%CI: 32.0–36.8) to 31.1% (95%CI: 29.8–32.4), respectively (Figure 1A). In contrast, a snapshot of the actual waiting lists on 31 December of each year revealed an average of 40.1% (95%CI: 39.2–41.0) of female patients.

### Reduced Transplantation Rates in Female Candidates

**FIGURE 2:**
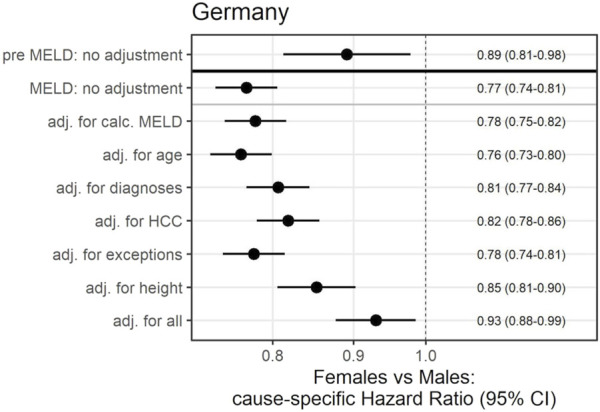
Effects of female sex on transplantation rates before and after the introduction of the MELD-based liver allocation system using competing-risk Cox regression (outcome: time to transplantation). HCC, hepatocellular carcinoma; MELD, Model for End-Stage Liver Disease.

Cumulative incidence analysis revealed that the chances of transplantation are significantly lower for women than for men (Figure 1B). Prior to the MELD era (pre-MELD), in waitlisted women had a slightly lower hazard of LT than men (HR = 0.89, 95%CI: 0.81–0.98). However, in the time-to-event model for the period after the MELD-based allocation system was introduced, the hazard of transplantation for women was estimated to be even lower, i.e., only 0.77-fold when compared with that of men (HR = 0.77, 95%CI: 0.74–0.81; Figure 2). Depending on the reference baseline, the absolute number of the gap since the introduction of MELD-based allocation until 2017, would be up to 731 transplants not allocated to women corresponding to approximately every 12th transplantation during this period (Supplementary Figure S3).

For better understanding, the analysis was adjusted for different covariates (Figure 2). Prior to the implementation of the MELD-based allocation system, sex-based discrimination could be explained by differences in height (Supplementary Figure S4). In the present allocation policy, differences between women and men in access to LT could be partially explained by adjustments for height (HR = 0.85, 95%CI: 0.81–0.90) as well as HCC, the most prevalent diagnosis for exceptional MELD (HR = 0.82, 95%CI: 0.78–0.86) (Figure 2).

### Waiting List Mortality and Survival After Transplantation

The MELD-based allocation system was implemented to reduce prolonged waiting times and mortality rates among patients on the waiting list. When examining waiting list mortality independently using competing risk regression with a cause-specific hazard, no significant sex differences were observed before the system’s introduction. Following the adoption of the MELD-based allocation system, women exhibited a slightly lower cause-specific hazard ratio for waiting list mortality (before HR = 0.96, 95%CI: 0.85–1.08; after HR = 0.86, 95%CI: 0.81–0.91) (Supplementary Figure S5). However, this statistic only captures the instantaneous effect on waiting list mortality. Overall, the reduced ratio does not result in a substantially lower overall waiting list mortality due to the adverse impact on the likelihood of transplantation. As a result, comparable rates of women and men die while waiting for liver transplantation or are removed from the waiting list for becoming unfit for transplantation (2 years after listing: 23.9% of female recipients; 24.3% of male recipients) (Figure 1B). Height was found to negatively influence waiting list mortality in women (Supplementary Figure S5). Survival after liver transplantation was comparable in the short term for men and women (1-year patient survival HR = 1.02, 95%CI: 0.92–1.12). In the longer term, female transplant recipients showed slightly better survival compared to men. This effect was already found in the pre-MELD era and did not significantly change thereafter (overall survival in women compared to men, pre-MELD HR = 0.85, 95%CI: 0.75–0.96; MELD HR = 0.89, 95%CI: 0.83–0.96) (Supplementary Figure S6).

### Calculated MELD and Serum Creatinine Withhold MELD Points in Women

**FIGURE 3:**
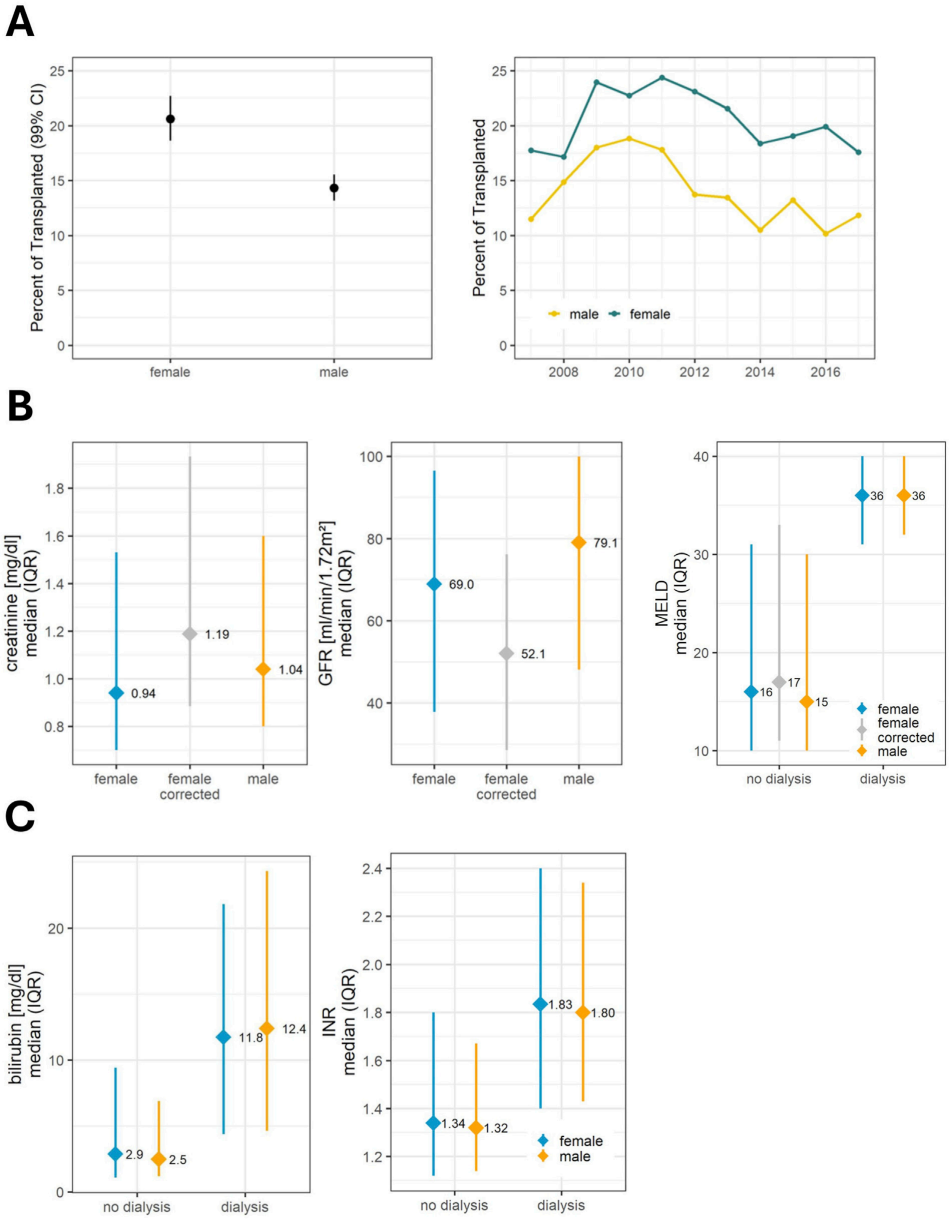
Components of the MELD score and influence of renal function. **(A)** Dialysis: Proportion of patients requiring dialysis at the time of organ allocation and its development over time. **(B)** Renal function and MELD: For patients without the need for renal replacement therapy, female recipients had lower serum creatinine values, but their actual renal function was worse than that of men. A corrected creatinine was used for the MELD calculation. For patients with a need for renal replacement therapy, the creatinine value in the MELD formula was set at 4 mg/dL. In this group, MELD scores do not differ between the sexes. **(C)** Further components of the MELD score: In the non-dialysis group bilirubin and INR were higher in the female cohort to compensate for lower creatinine. In the dialysis group, this was not the case. For the analysis of the MELD score, only the calculated MELD score was used without considering exceptional MELD. GFR, glomerular filtration rate (calculated using the CKD-EPI formula); INR, International normalized ratio; MELD, Model for End-Stage Liver Disease.

Reflecting the differences in transplantation rates by sex, no difference was observed in the calculated MELD score of all candidates at the time point of listing between men (median 17, IQR 10–29) and women (median 17, IQR 10–30). The MELD score of patients who actually received a transplant was higher in women (median 19, IQR 1–32) compared to men (median 17, IQR 11–30). To better understand this difference, the specific laboratory values that define the calculated MELD score were subjected to detailed analysis (Figure 3). Of particular interest is sCr as it is used without adaptation to well-known sex-based differences [10]. The cohorts were separated into recipients with and without renal replacement therapy; as for MELD score calculation sCr was set to 4 mg/dL in patients receiving dialysis. Overall, among patients who received a transplant, women were more likely to be on dialysis (20.6% vs. 14.3%, Figure 3A). Women who were transplanted without receiving renal replacement therapy had significantly lower sCr values than men (0.94 vs. 1.04 mg/dL; 99%CI = 0.90–0.99 vs. 1.01–1.07, Figure 3B), although their actual kidney function, represented by the eGFR, was significantly worse (52.1 vs. 69.0 mL/min; 99%CI = 49.7–54.4 vs. 65.8–72.0). Corrected sCr levels in women were higher than uncorrected levels (0.94 vs. 1.19 mg/dL; 99%CI = 0.90–0.99 vs. 1.14–1.25) and importantly those corrected sCR levels in women were higher compared to uncorrected levels in men (1.19 mg/dL vs. 1.04 mg/dL; 99%CI = 1.14–1.25 vs. 1.01–1.07). Subsequently, also women’s corrected MELD scores were higher compared to men’s. As an indicator of the need for women to compensate for this sex-unspecific use of sCr, female patients not on dialysis had increased levels of tBil and INR compared to men (tBil: women 2.9 mg/dL, 99%CI = 2.60–3.20, Figure 3C; men 2.5 mg/dL, 99%CI = 2.38–2.65; INR: women 1.34, 99%CI = 1.3–1.38; men 1.32, 99%CI = 1.30–1.34; Figure 3C). In a model, using a corrected MELD score, based on the eGFR corrected sCR levels as described above, women would gain up to three critical MELD points (Figure 3B; Supplementary Figure S7). Notably, additional MELD points would be assigned to all women with an eGFR below 85 mL/min. This would include 63.7% of female candidates who do not require renal replacement therapy. In a MELD-based allocation system missing MELD points could tip the balance and lead to lower chances of transplantation and longer waiting times (Supplementary Figure S8).

### Height-Related Hazards Disadvantage Shorter Candidates

**FIGURE 4:**
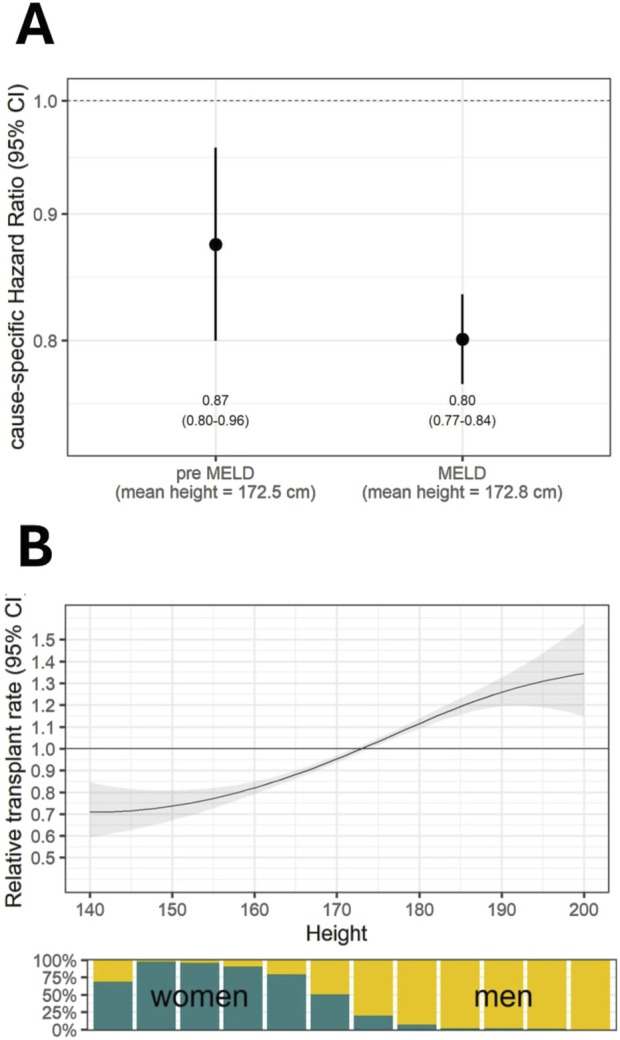
**(A)** Effects of height on transplantation rates using competing-risk Cox regression models. **(B)** Under the MELD-based allocation policy, the chances of transplantation increase directly with body height. The bar graph indicates the percentage of women in defined height groups.

By analyzing the hazard of height, we found that pre-existing height discrimination regardless of sex (HR = 0.87, 95%CI: 0.80–0.96) was exacerbated after implementation of the MELD-based liver allocation system (HR = 0.80, 95%CI: 0.77–0.84) (Figure 4A). This effect was found to be directly proportional to the height of the candidates with women constituting the vast majority of short individuals (Figure 4B).

### Exceptional MELD and Its Impact on Sex Inequity

**FIGURE 5:**
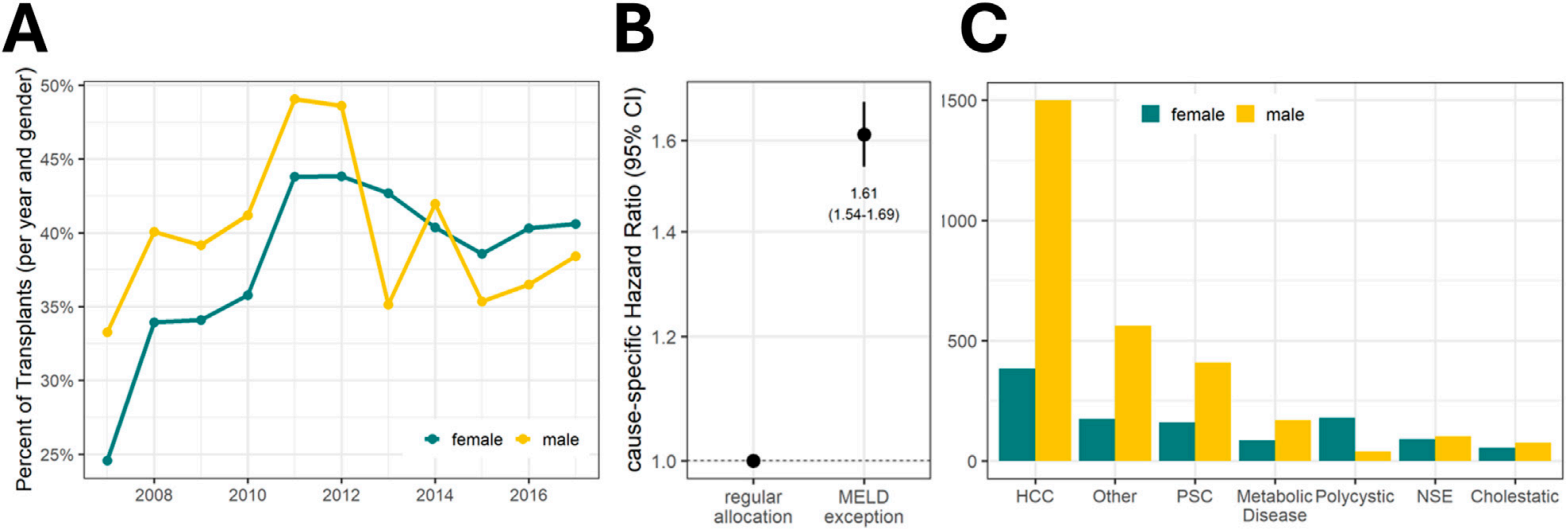
**(A)** Development of exceptional MELD over time. The percentage of exceptional MELD among transplant recipients increased after its implementation to nearly 50% in 2011 (43.8% for female recipients and 49.1% for male recipients). Overall, the share was higher in men than in women (40.2% vs. 37.5%). **(B)** Transplant probability of candidates by exceptional MELD status. **(C)** Indications for receiving exceptional MELD and their sex ratio. HCC, Hepatocellular Carcinoma; MELD, Model for End-Stage Liver Disease; NSE, Nonstandard Exception; PSC, Primary Sclerosing Cholangitis.

Certain indications are eligible for exceptional MELD points according to country-specific allocation guidelines. Consequently, the proportion of transplants based on these indications has increased over time (Figure 5A). Overall, 39.3% of effectively transplanted patients had a MELD exception. Men were more likely to receive an exceptional MELD (40.2% vs. 37.5% in women, Supplementary Table S2) and candidates with an exceptional MELD have a higher chance of undergoing transplantation compared to those without (HR = 1.61, 95% CI: 1.54–1.69; Figure 5B). The most frequent diagnosis for the standardized exceptional MELD is HCC and in the group of patients receiving an exceptional MELD for this reason the number of women is disproportionately low (Figure 5C).

## Discussion

This is the first study to demonstrate that the introduction of a MELD-based liver allocation system has exacerbated an existing disadvantage in the chances of women undergoing deceased donor LT in Germany. Although for the United States, this has been indicated previously [2, 3, 12, 13], very few comparable data are available for other countries [14]. Based on the large cohort data, the similarity of the allocation systems and the identified systematic flaws, we believe that this inequity is of relevance in all countries using similar MELD-based allocation systems [15].

Globally, of the more than 1,3 million deaths per year due to cirrhosis, the proportion of women dying due to liver failure is one-third [16] and the risk of liver-related death is similar to that of men [17]. Despite stable sex proportions of liver-related causes of death, we observed an increase in the effective male-to-female LT ratio over time, to the detriment of women. Certainly, liver-related mortality does not necessarily match exactly with the incidence, prevalence, or burden of end-stage liver disease nor does it implicitly correspond to the indication for LT. However, the HEPAHEALTH project by the European Association for the Study of the Liver [18] and the Global Burden of Disease Study [16] have revealed that the aforementioned sex ratio of liver-related deaths matches the epidemiology of liver disease and that the ratio has remained constant over time. Based on our data, the disadvantage of women undergoing liver transplantation is based on four aspects, namely, reduced waitlisting, calculated MELD, height, and exceptional MELD.

First, women are disproportionately less likely to be listed for liver transplantation. This imbalance may be based on the disadvantages caused by serum sCr and exceptional MELD, as the majority of transplant centers implemented absolute MELD thresholds for waitlisting and/or transplantation [19].

Second, female recipients had lower values of sCr even though their actual renal function was worse than in male patients (Figure 5; Supplementary Figure S9). This difference can account for up to three or even more MELD points [9, 20]. Because of the lower muscle mass of women, they have lower sCr levels [21] which heavily affect the MELD score [22]. The implementation of MELD-Na in the United States in 2016 has exacerbated this disparity [20]. As we have shown, a woman has to be “sicker” to have the same MELD score as a man, which explains the higher waitlist frailty, mortality, and dropout rate due to ineligibility for LT previously described in women [22, 23].

Third, height is lower in women, which has a negative impact on the probability of receiving LT. Some studies have already highlighted that lower body height in women is associated with a higher waiting list mortality in the United States [20, 24–26]. Although there is no solid evidence of how height affects the chances of organ allocation in this objective system, the most obvious reason is a decrease in organ offers due to fear of large-for-size syndrome [27, 28]. Consequently, the complete spectrum of offered donor livers is accepted in terms of organ volume for male recipients, whereas only a portion is accepted for female recipients [29]. In a recent study this difference in acceptance of organ offers was found to persist even in patients with high disease severity, resulting in a lower chance of receiving a transplant and a higher waiting list mortality rate [30]. Therefore, size compensation may be needed for retributive justice.

Fourth, women are less likely to receive an exceptional MELD. It is known that patients with exceptional MELD are generally more likely to receive LT and have lower waiting list mortality [31, 32]. The most common standardized exception, HCC, is more frequent in men [33]. In the German transplant registry, only 20.4% of all HCC patients were female. In the United States, the rules for HCC exceptions have already been adapted and revised in 2009 and 2015 to address the imbalance between HCC and non-HCC patients.

Consequently, it is essential to optimize current allocation systems worldwide to address these sex inequities. To compensate for the loss of MELD score points due to the use of sCr, either additional MELD score points for women [9, 20], a corrected sCr [34], or the implementation of GFR into the MELD formula have been suggested, partially demonstrating a harmonization of waiting list mortality [35–37]. Our study suggests that the recipient’s height should also be considered to counteract the problem of large-for-size [24, 29]. Organs from shorter donors could be allocated preferentially to shorter recipients (regardless of sex) or small people could otherwise receive extra MELD score points as in pediatric transplantation [6, 38]. Finally, exceptional MELD status can be adjusted by policy changes, e.g., reduction of exceptional points. In the United States, following a growing debate [39], the first specific policy modification was adopted in 2023 to minimize sex-based differences by using the so-called MELD 3.0 which assigns an additional 1.33 MELD points to women and adjusts the limits of included laboratory values [7]. This represents a crucial step in addressing the disparities also identified in our study. However, the specific effect of this adjustment remains to be investigated, as the described factors such as height and exceptional MELD are not explicitly addressed. In other countries, such adjustments are still lacking, and data from outside the United States are largely insufficient to justify such modifications. Although MELD-based allocation is utilized globally, significant differences persist in transplant and healthcare systems. There are notable disparities in organ donation availability, the exact design of MELD-based allocation (e.g., criteria for exceptional MELD points), and financial and socio-economic access to transplantation. Therefore, it is essential to consider and analyze local contexts and potentially tailor guidelines to meet specific regional circumstances. Recently, Tejedor et al. published their findings from an analysis of the Spanish Liver Transplantation Registry [40]. Their study represents the first national investigation outside the United States on this topic and also found lower transplantation rates for women compared to men. Spain and the United States have the highest rates of organ donation internationally and utilize a significant number of donations after circulatory death. Although the Spanish study is an important step, the applicability of the existing findings to many other countries remains uncertain. Our study helps to fill this knowledge gap: Germany, with an average organ donation rate and no current practice of transplanting organs from donors after circulatory death, is more representative of many other countries than Spain and the United States. The fact that we found similar results suggests that sex-based inequity is inherent in the system, highlighting the need for a global discussion and adaptation of allocation rules.

Regarding waiting list mortality, we described comparable absolute waiting list mortality rates for women and men, but we also reported a reduction in the cause-specific hazard ratio for waiting list mortality for women. This may seem contradictory and inconsistent with previous reports from the United States [2, 3, 12]. However, in the present study an effective reduction in waiting list mortality was probably not achieved due to the adverse effect on the likelihood of transplantation. Differences with previous reports may have been influenced by the statistic selected to analyze the competing risks of transplantation and waiting list mortality. In this context, it is also reasonable to assume that the analysis of waiting list mortality is always complicated by changes in allocation policies as waiting list registrations are highly dependent on the chances of transplantation and the majority of transplant centers will only evaluate patients who meet certain criteria (e.g., threshold of MELD score, exceptional MELD). Therefore, a change in allocation rules will alter the listing behavior of transplant centers. The resulting shift in the composition of the waiting list makes direct comparisons challenging. This confirms our approach of additionally relating the sex ratio in the transplant system to the entire patient population.

The quality of our results depends on the quality of data entry. All data shown are analyzed retrospectively and therefore do not provide proof of any causal relationships, although the evidence seems clear. However, these limitations are comparable to similar studies.

In conclusion, women in need of LT face two problems: they are less likely to be waitlisted, and their chances of receiving a transplant are lower than those of men. Although the implementation of a MELD-based liver allocation system aimed to guarantee a fair and objective organ allocation, this was not accomplished in terms of sex-based equity. As the results of our study are in line with other international studies, this sex-based inequity must be resolved worldwide. Possible approaches to improve the allocation system would be to consider the inclusion of the height of the recipients, a reevaluation of the renal function, and a discussion of the priority of patients with HCC in all MELD-based transplantation programs.

## Data Availability

The data analyzed in this study is subject to the following licenses/restrictions: Data is not directly available from the researchers but may be requested from the working group for liver transplantation at the German Medical Association (Bundesärztekammer). Alternatively data may be requested from the German transplant registry. Requests to access these datasets should be directed to https://transplantations-register.de/.

## Abbreviations

LT, liver transplantation; MELD, Model for End-stage Liver Disease; tBil, total bilirubin; sCr, serum creatinine; INR, international normalized ratio; HCC, hepatocellular carcinoma; eGFR, estimated glomerular filtration rate; IQR, interquartile range; HR, hazard ratio; CI, confidence interval.
