# Supplementary material for: Systematic Sex-Based Inequity in the MELD Score-Based Allocation System for Liver Transplantation in Germany
Source: Transpl Int. 2025 Jan 29;38:13844. doi: 10.3389/ti.2025.13844 (PMC11813689; doi:10.3389/ti.2025.13844)
Supplement: Supplementary file 1 [file DataSheet1.docx]

**Supplementary Material**

**Capsule Sentence Summary** 3

**Supplementary Methods**

1. Definitions of different types of MELD score 4

2. Standardized MELD Exceptions 5

3. Calculations 6

4. Liver-related cause of death 7

5. Used statistical software 8

**Supplementary Tables**

Table S1. Demographics of liver candidates 9

Table S2. Demographics of liver recipients 10

Table S3. Comparison of deviating data source (preMELD era) 11

Table S4. Comparison of deviating data source (MELD era) 12

**Supplementary Figures**

Figure S1. Flow chart of patient inclusion 13

Figure S2. Development of sex proportions 14

Figure S3. Sex gap in liver transplantation 15

Figure S4. Female transplant probability and transplant rates

(preMELD era) 17

Figure S5. Waiting list mortality 19

Figure S6 Survival after transplantation 20

Figure S7 Corrected MELD score accordingly to renal function 21

Figure S8. Association of MELD score at listing with transplant rate 22

**References** 23

**Capsule Sentence Summary**

MELD-based liver allocation aggravated sex disparities in Germany, with women less likely to be waitlisted or transplanted due to height differences, kidney function measurement and MELD exceptions, highlighting the need for fair and sex-neutral allocation practices."

**Supplementary Methods 1. Definitions of different types of MELD score**

| **Nomenclature used in this study** | **Equivalent nomenclature** | **Definition** |
| --- | --- | --- |
| Calculated MELD score* | Laboratory MELD score (“labMELD”) | Score calculated using the MELD formula including serum creatinine, total bilirubine and INR; ranges from 6 to 40* |
| Exceptional MELD score | Standardized exception MELD score (“seMELD”) or  Non-standardized exception MELD score (“nseMELD”) | Score that can be assigned to patients if their calculated MELD score does not appropriately reflect their urgency for transplant;   - Standardized MELD exceptions are provided for defined indications if certain requirements are fulfilled - Non-standardized MELD exceptions can be provided upon request for patients outside the predefined rules |
| Allocation MELD score | matchMELD score | The highest MELD score available to a candidate according to policy at the time of a match–could be calculated or exception MELD score |

**Supplementary Methods 2. Standardized MELD Exceptions**

Standardized MELD exceptions—representing diseases that regularly receive additional MELD points for allocation, as their severity is not adequately reflected by the calculated MELD score—are currently defined for the following indications:

In Germany, MELD exceptions are foreseen for cholangiocarcinoma, cystic fibrosis, familial amyloid polyneuropathy, hepatic artery thrombosis after liver transplantation, hepatopulmonary syndrome, metabolic disease (urea cycle disorder or organic academia), portopulmonary hypertension, primary hyperoxaluria, and hepatocellular carcinoma (HCC) as well for polycystic liver disease, persistent hepatic dysfunction after liver transplantation (formerly “small for size” syndrome), hereditary hemorrhagic telangiectasia, hepatic hemangioendothelioma, biliary sepsis, secondary sclerosing cholangitis, primary sclerosing cholangitis, and neuroendocrine tumors.

Additionally, non-standardized MELD exceptions can be provided upon request for patients if their calculated MELD score does not appropriately reflect their urgency for transplant but they are outside the predefined rules.

**Supplementary Methods 3. Calculations**

*MELD score^1^*

calculated MELD score = 0.957 x Log_e_ (creatinine mg/dL) + 0.378 x Log_e_ (bilirubin mg/dL) + 1.120 x Log_e_ (INR) + 0.643

*Estimated glomerular filtration rate*

chronic kidney disease epidemiology (CDK-EPI) formula^2^

GFR = 141 × min(Scr/κ, 1)α × max(Scr/κ, 1)-1.209 × 0.993Age × 1.018 [if female] _ 1.159 [if black]

Scr = serum creatinine

κ = 0.7 (females) or 0.9 (males)

α = -0.329 (females) or -0.411 (males)

*Corrected serum creatinine for females*

eGFR = 141 × min(**corScr**/κ, 1)α × max(**corScr**/κ, 1)-1.209 × 0.993Age × 1.159 [if black]

[formula resolved for corScr]

eGFR = calculated eGFR of females using the above described formula

corScr = corrected serum creatinine

κ = 0.9

α = -0.411

*corrected MELD score*

corrected MELD score = 0.957 x Log_e_ (**corScr** mg/dL) + 0.378 x Log_e_ (bilirubin mg/dL) + 1.120 x Log_e_ (INR) + 0.643

corScr = corrected serum creatinine (derived from formula above)

**Supplementary Methods 4. Liver-related causes of death**

To define a baseline of sex-specific differences in end-stage liver disease, we analyzed sex-specific liver-related mortality statistics.

Liver-related causes of death were defined as death due to viral hepatitis (ICD-10: B15–B19), malignant neoplasms of the liver and intrahepatic bile ducts (C22), and all other diseases of liver (K70–K76).

Data were obtained from the Federal Statistical Office.

**Supplementary Methods 5. Used statistical software**

Used software: R^3^

Additional packages for data handling and plotting: Tidyverse^4^, cmprsk^5^, ggplot2^6^

Additional packages for Cox regression: survival^7,8^

**Table S1. Demographics of liver candidates.**

|  | **preMELD***  **(2003–2007)** | | **MELD**  **(2007–2017)** | | **Total** |
| --- | --- | --- | --- | --- | --- |
|  | **Male**  n = 2,219 | **Female**  n = 1,143 | **Male**  n = 10,980 | **Female**  n = 5,676 | n = 20,018 |
| **Age** | 54.0 (47.0–60.0) | 53.0 (45.0–60.0) | 56.0 (50.0–62.0) | 55.0 (48.0–62.0) | 56.0 (49.0–62.0) |
| **BMI** | 25.6 (23.1–28.6) | 23.7 (21.3–26.9) | 26.2 (23.5–29.4) | 24.2 (21.5–28.10) | 25.5 (22.6–29.0) |
| **Height** | 176 (172–181) | 165 (160–169) | 177 (172–182) | 165 (160–169) | 173 (167–180) |
| **Calculated MELD** |  |  | 17 (10–29) | 17 (10–30) | 17 (10–29) |
| **Allocation MELD** |  |  | 22 (12–31) | 21 (10–32) | 21 (11–31) |
| **MELD exception** |  |  | 2,312 (21.1%) | 973 (17.1%) | – |
| **Dialysis** | 38 (1.7%) | 18 (1.6%) | 1,598 (14.6%) | 1,009 (17.8%) | 2,663 (13.3%) |
| **Diagnosis**  Acute hepatic necrosis  Cholestatic liver disease  Noncholestatic cirrhosis  Malignant  neoplasm  Other | 41  (1.8%)  163  (7.3%)  935  (42.1%)  355  (16.0%)  725  (32.7%) | 23  (2.0%)  157  (13.7%)  451  (39.5%)  90  (7.9%)  422  (36.9%) | 347  (3.2%)  1,140  (10.4%)  5,485  (50.0%)  3,343  (30.4%)  665  (6.1%) | 273  (4.8%)  786  (13.8%)  2,890  (50.9%)  946  (16.7%)  781  (13.8%) | 684  (3.4%)  2,246  (11.2%)  9,761  (48.8%)  4,734  (23.6%)  2,060  (10.3%) |
| **Waiting time** | 145 (55–347) | 153 (55–416) | 206 (49–630) | 293 (57–929) | 210 (53–629) |

Data presented as median and interquartile range or count and percentage

*Defined as candidates listed and delisted before introduction of MELD-based allocation

*BMI, Body Mass Index; MELD, Model for End-Stage Liver Disease*

**Table S2. Demographics of liver recipients.**

|  | **preMELD**  **(2003–2006)** | | | **MELD**  **(2007–2017)** | | **Total** |
| --- | --- | --- | --- | --- | --- | --- |
|  | **Male**  n = 1,530 | | **Female**  n = 771 | **Male**  n = 5,856 | **Female**  n = 2,647 | n = 10,804 |
| **Age** | 53.0 (46.0–59.0) | | 52.0 (44.0–60.0) | 56.0 (49.0–61.0) | 54.0 (47.0–61.0) | 55.0 (48.0–61.0) |
| **BMI** | 25.6 (23.2–28.4) | | 23.6(21.1–26.7) | 26.3 (23.6–29.4) | 24.2 (21.5–27.8) | 25.6 (22.8–29.0) |
| **Height** | 176 (172–182) | | 165 (160–170) | 178 (172–182) | 165 (160–170) | 174 (168–180) |
| **Calculated MELD** |  | |  | 17 (11–30) | 19 (11–32) | – |
| **Allocation MELD** |  | |  | 26 (18–32) | 28 (20–34) | – |
| **MELD exception** |  | |  | 2,353 (40.2%)^1^ | 993 (37.5%)^1^ | – |
| **Dialysis** |  | |  | 839 (14.3%) | 546 (20.6%) | – |
| **Serum creatinine^**^** |  | |  | 1.1 (0.8–1.9)^1^ | 1.0 (0.7–1.8)^1^ | – |
| **Total bilirubin** |  | |  | 2.9 (1.3–9.9)^1^ | 3.8 (1.2–13.1)^1^ | – |
| **INR** |  | |  | 1.4 (1.2–1.8)^1^ | 1.4 (1.2–2.0)^1^ | – |
| **Diagnosis**  Acute hepatic necrosis  Cholestatic liver disease  Noncholestatic cirrhosis  Malignant  neoplasm  Other | 14  (0.9%)  131  (8.6%)  694  (45.4%)  243  (15.9%)  448  (29.3%) | | 11  (1.4%)  130  (16.9%)  322  (41.8%)  54  (7.0%)  254  (32.9%) | 156  (2.7%)  658  (11.2%)  3123  (53.3%)  1542  (26.3%)  377  (6.4%) | 106  (4.0%)  421  (15.9%)  1280  (48.4%)  428  (16.2%)  412  (15.6%) | 287  (2.7%)  1340  (12.4%)  5419  (50.2%)  2267  (21.0%)  1491  (13.8%) |
| **Split organ** | 75 (4.9%)^1^ | | 55 (7.1%)^1^ | 164 (2.8%)^1^ | 182 (6.9%)^1^ | 476 (4.4%) |
| **CIT** | 9.9 (8.2–11.2)^1^ | | 9.8 (8.2–11.2)^1^ | 9.4 (7.8–11.0)^1^ | 9.3 (7.8–10.9)^1^ | 9.5 (7.9–11.0) |
| **Waiting time** | 186 (69–354) | | 231 (83–440) | 137 (31–347) | 159 (29–452) | 155 (39–377) |
| **Donor characteristics** | |  |  |  |  |  |
| **Age** | 51.0 (40.25–62.0)^1^ | | 49.0 (37.0–62.0)^1^ | 56.0 (45.0–67.0)^1^ | 54.0 (43.0–67.0)^1^ | 54.0 (43.0–66.0) |
| **Male sex**  **Female sex** | 943 (61.6%)^1^  587 (38.4%)^1^ | | 327 (42.4%)^1^  444 (57.6%)^1^ | 3,515 (60.0%)^1^  2,341 (40.0%)^1^ | 1,062 (40.1%)^1^  1,585 (59.9%)^1^ | 5,847 (54.1%)^1^  4,957 (45.9%)^1^ |
| **BMI** | 25.6 (23.5–27.8)^1^ | | 24.2 (22.5–26.2)^1^ | 26.2 (24.2–29.3)^1^ | 24.7 (22.9–27.6)^1^ | 25.7 (23.5–27.8) |

Data presented as median and interquartile range or count and percentage

**set to 4 mg/dl if patient was on dialysis (according to MELD formula)

^1^due to data quality reasons, the number of patients in the cohort of the marked values may differ (see Table S5 and S6)

*BMI, Body Mass Index; CIT, Cold Ischemic time; MELD, Model for End-Stage Liver Disease*

**Table S3. Comparison of deviating data source for data in Table S2 (preMELD era).**

| **Liver recipients before MELD era (2003–2006)** | | | | | | | | | |
| --- | --- | --- | --- | --- | --- | --- | --- | --- | --- |
| **Data source** | | ***Waiting list database*** | | | | ***Transplant database*** | | | |
|  | | **Male**  n = 1343 | | **Female**  n = 666 | | **Male**  n = 1530 | | **Female**  n = 771 | |
| **Deviation** | | – | | – | | 12.3% | | 13.6% | |
| **Age** | | 53.0 (46.0–59.0) | | 52.5 (44.0–60.0) | | 53.0 (46.0–59.0) | | 52.0 (44.0–60.0) | |
| **BMI** | | 25.6 (23.3–28.6) | | 23.7 (21.5–26.9) | | 25.6 (23.2–28.4) | | 23.6(21.1–26.7) | |
| **Height** | | 177 (172–182) | | 165 (160–170) | | 176 (172–182) | | 165 (160–170) | |
| **Dialysis** | | No data | | – | | – | | – | |
| **Diagnosis**  Acute hepatic necrosis  Cholestatic liver disease  Noncholestatic cirrhosis  Malignant  neoplasm  Other | | 10  (0.7%)  115  (8.6%)  553  (41.2%)  231  (17.2%)  434  (32.3%) | | 7  (1.1%)  113  (17.0%)  238  (35.7%)  46  (6.9%)  262  (39.3%) | | 14  (0.9%)  131  (8.6%)  694  (45.4%)  243  (15.9%)  448  (29.3%) | | 11  (1.4%)  130  (16.9%)  322  (41.8%)  54  (7.0%)  254  (32.9%) | |
| **Split organ** | | No data | | – | | 75 (4.9%) | | 55 (7.1%) | |
| **CIT** | | No data | | – | | 9.9 (8.2–11.2) | | 9.8 (8.2–11.2) | |
| **Waiting time** | | 157 (62–342) | | 197 (73.75–424) | | 186 (69–354) | | 231 (83–440) | |
| **Donor characteristics** | | | | | | | | | |
| **Age** | No data | | – | | 56.0 (45.0–67.0) | | 54.0 (43.0–67.0) | |  |
| **Male sex**  **Female sex** | No data | | – | | 3,515 (60.0%)  2,341 (40.0%) | | 1,062 (40.1%)  1,585 (59.9%) | |  |
| **BMI** | No data | | – | | 26.2 (24.2–29.3) | | 24.7 (22.9–27.6) | |  |
| **Height** | No data | | – | | 175.2 (±9.2) | | 170.9 (±10.1) | |  |
| **Hypertension** | No data | | – | | 484 (31.6%) | | 228 (29.6%) | |  |

Data presented as median and interquartile range or count and percentage

*BMI, Body Mass Index; CIT, Cold Ischemic time; MELD, Model for End-Stage Liver Disease*

**Table S4. Comparison of deviating data source for data in Table S2 (MELD era).**

| **Liver recipients during MELD era (2007–2017)** | | | | |
| --- | --- | --- | --- | --- |
| **Data source** | ***Waiting list database*** | | ***Transplant database*** | |
|  | **Male**  n = 6,095 | **Female**  n = 2,767 | **Male**  n = 5,856 | **Female**  n = 2,647 |
| **Deviation** | - | - | 4.1% | 4.5% |
| **Age** | 56.0 (49.0–62.0) | 54.0 (49.0–62.0) | 56.0 (49.0–61.0) | 54.0 (47.0–61.0) |
| **BMI** | 26.3 (23.7–29.5) | 24.4 (21.6–28.0) | 26.3 (23.6–29.4) | 24.2 (21.5–27.8) |
| **Height** | 178 (172–182) | 165 (160–170) | 178 (172–182) | 165 (160–170) |
| **Calculated MELD** | 17 (11–30) | 19 (11–32) | 17 (11–30) | 19 (11–32) |
| **Allocation MELD** | 25 (15–32) | 27 (16–34) | 26 (18–32) | 28 (20–34) |
| **MELD exception** | No data | – | 2,353 (40.2%) | 993 (37.5%) |
| **Dialysis** | 858 (14.7%) | 544 (20.6%) | 839 (14.3%) | 546 (20.6%) |
| **Serum creatinine^**^** | No data | – | 1.1 (0.8–1.9) | 1.0 (0.7–1.8) |
| **Total bilirubin** | No data | – | 2.9 (1.3–9.9) | 3.8 (1.2–13.1) |
| **INR** | No data | – | 1.4 (1.2–1.8) | 1.4 (1.2–2.0) |
| **Diagnosis**  Acute hepatic necrosis  Cholestatic liver disease  Noncholestatic cirrhosis  Malignant  neoplasm  Other | 148  (2.4%)  721  (11.8%)  2,639  (43.3%)  2,190  (35.9%)  397  (6.5%) | 103  (3.7%)  442  (16.0%)  1,173  (42.4%)  605  (21.9%)  444  (16.0%) | 156  (2.7%)  658  (11.2%)  3,123  (53.3%)  1,542  (26.3%)  377  (6.4%) | 106  (4.0%)  421  (15.9%)  1,280  (48.4%)  428  (16.2%)  412  (15.6%) |
| **Split organ** | No data | – | 164 (2.8%) | 182 (6.9%) |
| **CIT** | No data | – | 9.4 (7.8–11.0) | 9.3 (7.8–10.9) |
| **Waiting time** | 144 (34–363) | 179 (33–482) | 137 (31–347) | 159 (29–452) |
| **Donor characteristics** | | | | |
| **Age** | No data | – | 56.0 (45.0–67.0) | 54.0 (43.0–67.0) |
| **Male sex**  **Female sex** | No data | – | 3,515 (60.0%)  2,341 (40.0%) | 1,062 (40.1%)  1,585 (59.9%) |
| **BMI** | No data | – | 26.2 (24.2–29.3)^1^ | 24.7 (22.9–27.6) |
| **Height** | No data | – | 27.1 (6.4) | 25.3 (4.2) |
| **Hypertension** | No data | – | 2340 (40.0%) | 948 (35.8%) |

Data presented as median and interquartile range or count and percentage

*BMI—Body Mass Index; CIT—cold ischemic time; MELD—Model for End-Stage Liver Disease.*

**Figure S1. Flow chart of patient inclusion.**

Period: 2003–2017

Patients registered on waiting list

(n = 25,943/ 37.4% ♀)

Analyzed waiting list patients

(n = 20,018/ 34.1% ♀)

Excluded (n = 5,925/ 48.6% ♀)

Age < 18 (n = 2,048/ 49.3% ♀)

Living donation (n = 1,016/ 44.6% ♀)

Combined organ transplantation—data not available

Status 1 (n = 3,151/ 51.3% ♀)

Pre MELD era

Removal 01/01/2003–12/31/2006

(n = 3,362/ 34.0% ♀)

MELD era

Removal 01/01/2007–12/31/2017

(n = 16,656/ 34.1% ♀)

Reasons for removal

Transplanted (n = 2,009/ 33.2% ♀)

Deceased/unfit for transplantation (n = 1,149/ 34.4% ♀)

Recovered (n = 21/ 47.6% ♀)

Other (n = 183/ 39.3% ♀)

Reasons for removal

Transplanted (n = 8,473/ 31.2% ♀)

Deceased/unfit for transplantation (n = 5,372/ 34.4% ♀)

Recovered (n = 1,514/ 45.9% ♀)

Other (n = 908/ 40.3% ♀)

Overall liver-related cause of death

***35***.***0% ♀***

**Figure S2. Development of sex proportions.**

The share of women in liver-related causes of death was quite stable over the studied period (33.7%–35.8%). By contrast, the proportion of women who were registered on the waiting list decreased after MELD implementation (preMELD 36.2%; MELD 33.3%). Similarly, a strong decrease could be observed in the proportion of actual female liver transplant recipients (preMELD 34.4%; MELD 31.1%). Data on causes of death were derived from the German Federal Statistical Office.

**Figure S3. Sex gap in liver transplantation.**

A.

C.

B.

****D.

To quantify the absolute gap of women in the current liver transplantation system we related the actual numbers of liver transplantation respectively actual listings to different reference baselines to estimate how many females would be missing to generate a balanced system (delta ∆).

1. Relation of waiting list registrations to liver-related causes of death
2. Relation of liver transplantations to liver-related causes of death.
3. Relation of liver transplantations to waiting list registrations.
4. Relation of liver transplantations to candidates on the waiting list at year-end.

**Figure S4. Female transplant probability and transplant rates in preMELD era.**


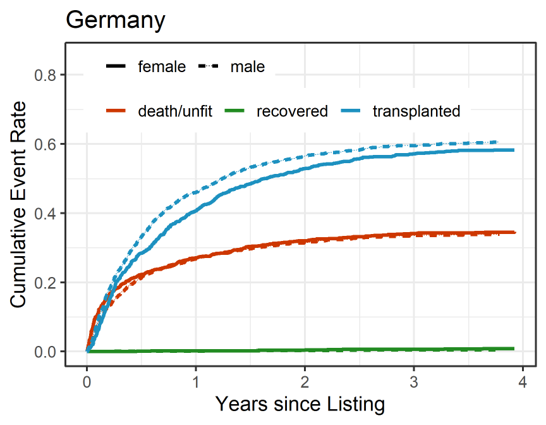
A–Outcome probability of candidates.

(A) Time-to-event analysis (cumulative incidence function) showed a slightly reduced chance to receive liver transplantation for women in the preMELD allocation systems. After two years on the transplant waiting list, the chance of getting transplanted is d 3.6% lower for women than for men. However, differences between women and men were less pronounced in comparison with the MELD era (Figure 1B).

**
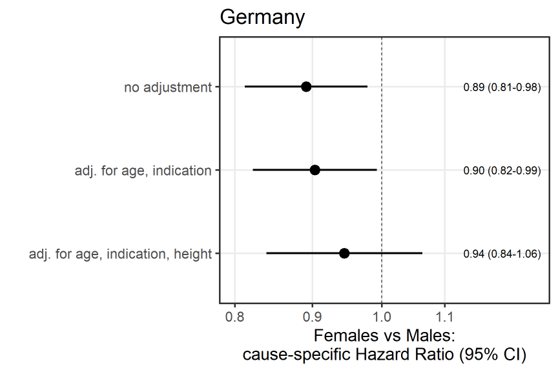
**B–Association of female sex with transplantation rates in preMELD era.

(B) Competing-risk Cox regression models (outcome: time to transplant) show that reduced transplant rates of female candidates in the preMELD era were mostly independent of age and indications but majority of the difference could be explained by differences in body height.

**Figure S5. Waiting list mortality.**


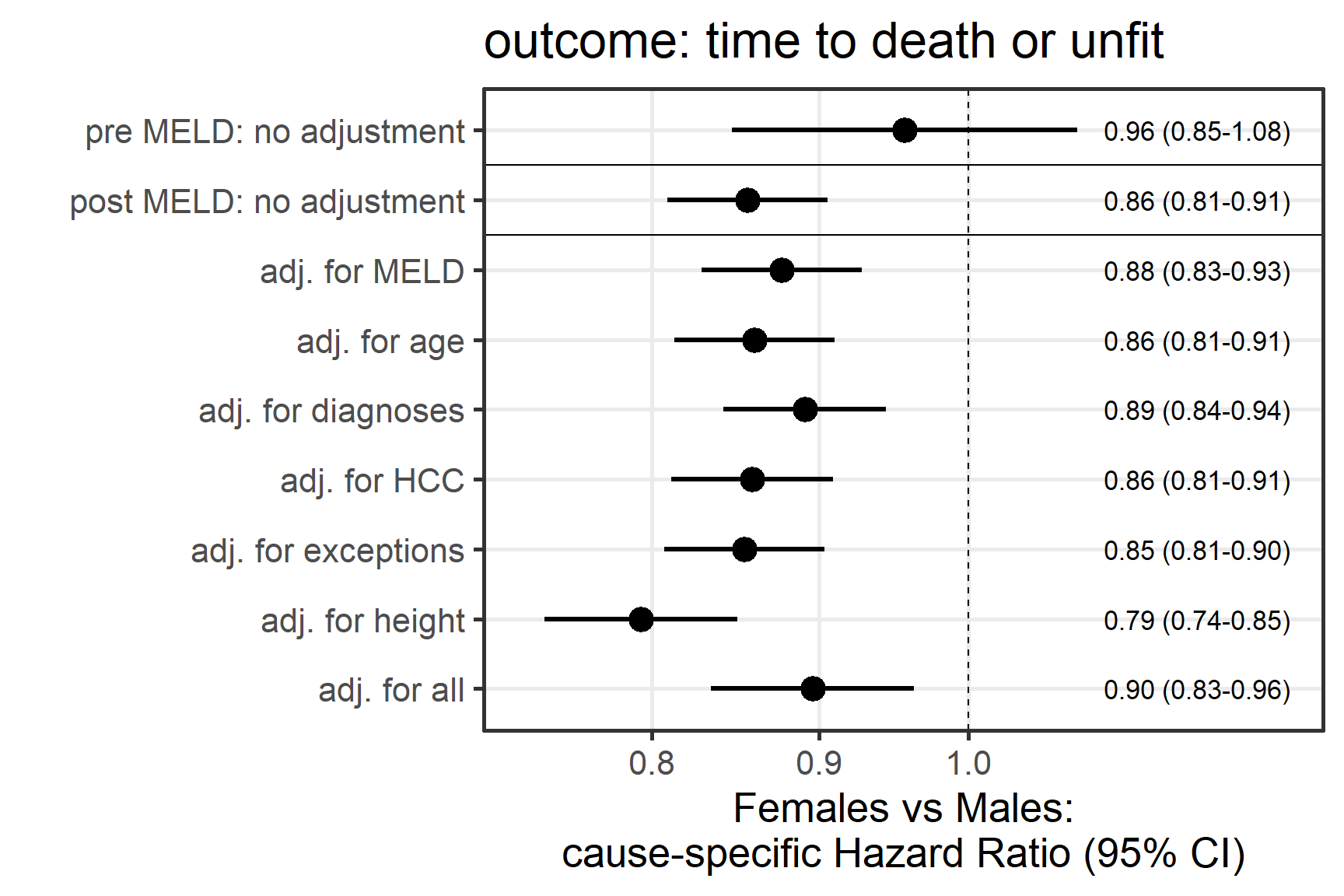


Association of female sex with waiting list mortality before and after the introduction of MELD-based liver allocation using competing-risk Cox regression (outcome: time to death or becoming unfit for transplantation).

*HCC—hepatocellular carcinoma; MELD—Model for End-Stage Liver Disease*

**Figure S6 Survival after transplantation**

A–Overall patient survival after liver transplantation.


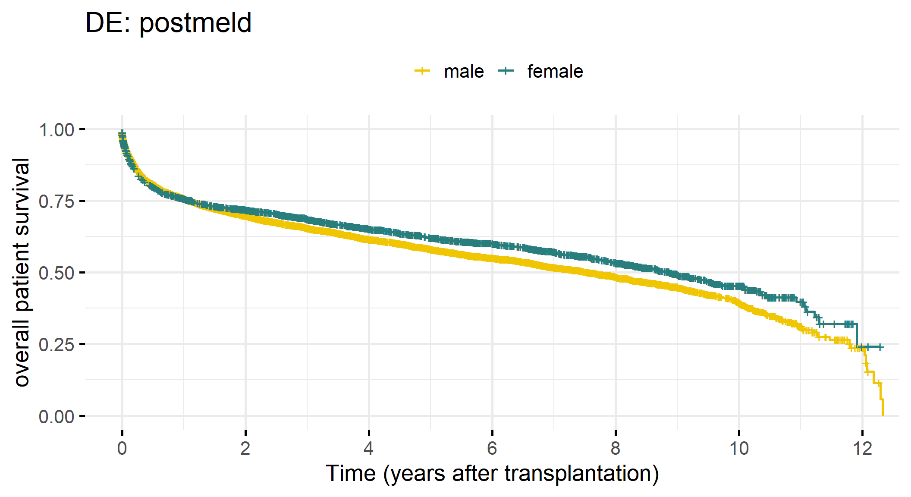


Germany


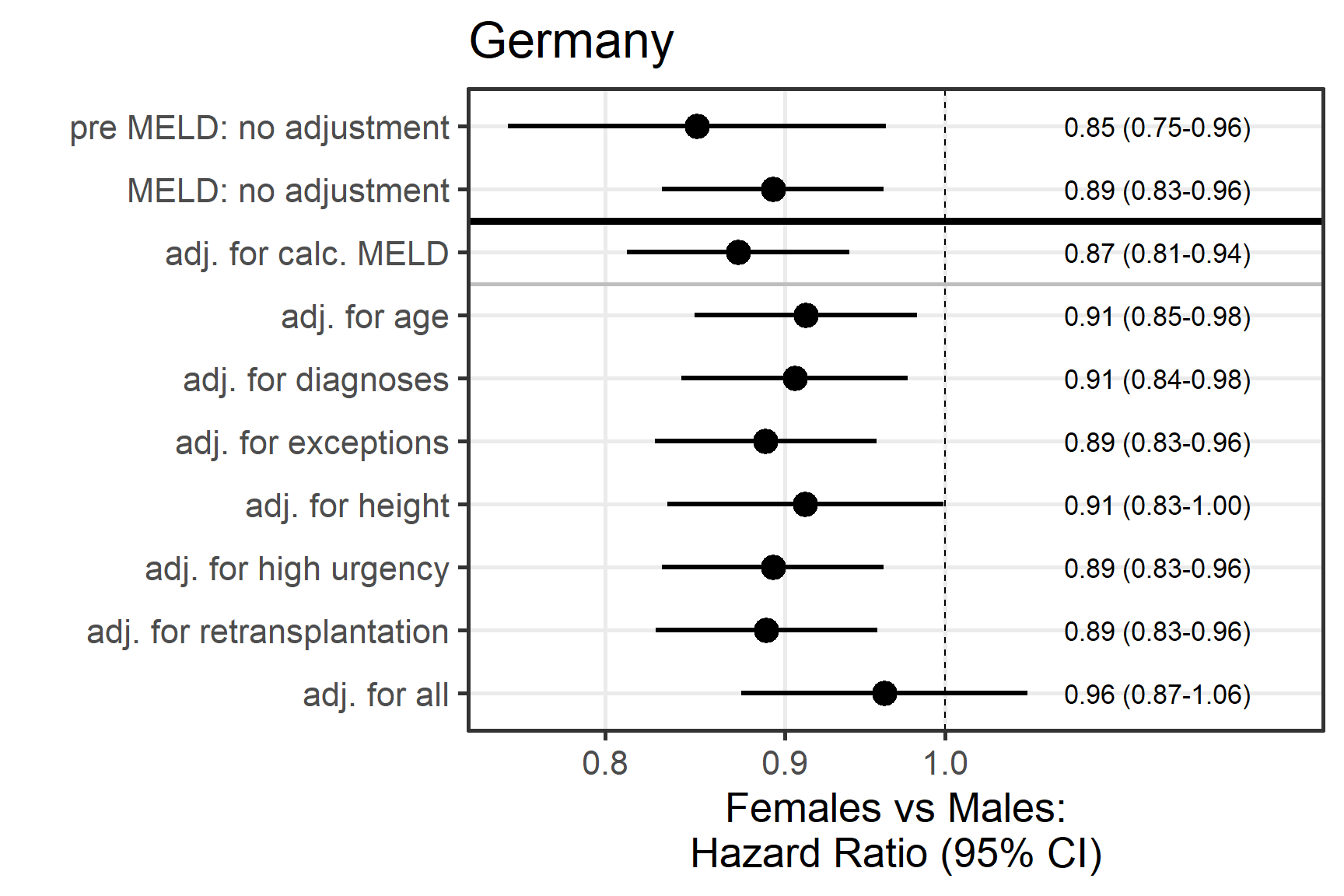
B– Association of female sex with overall patient mortality rates.

(A) Kaplan-Meier curves displaying overall patient survival after liver transplantation. Follow-up began at time of transplantation and ended at death or was censored at time of last documented follow-up.

(B) The association of female sex with mortality after transplantation before and after the introduction of MELD-based liver allocation were analyzed using Cox regression (outcome: overall patient mortality).

*HCC—hepatocellular carcinoma; MELD—Model for End-Stage Liver Disease*

**Figure S7 Corrected MELD score accordingly to renal function.**

By using a corrected creatinine value, a corrected MELD score was calculated for female recipients. The corrected value corresponds to the creatinine value, which represents the actual renal function by inserting the actual GFR of women into the male formula for GFR (as creatinine is inserted without correction for sex in the MELD formula). The median difference between actual MELD and corrected MELD is one point (range 0–3).

*GFR—Glomerular Filtration Rate (Calculated Via CKD-EPI Formula); MELD—Model for End-Stage Liver Disease*


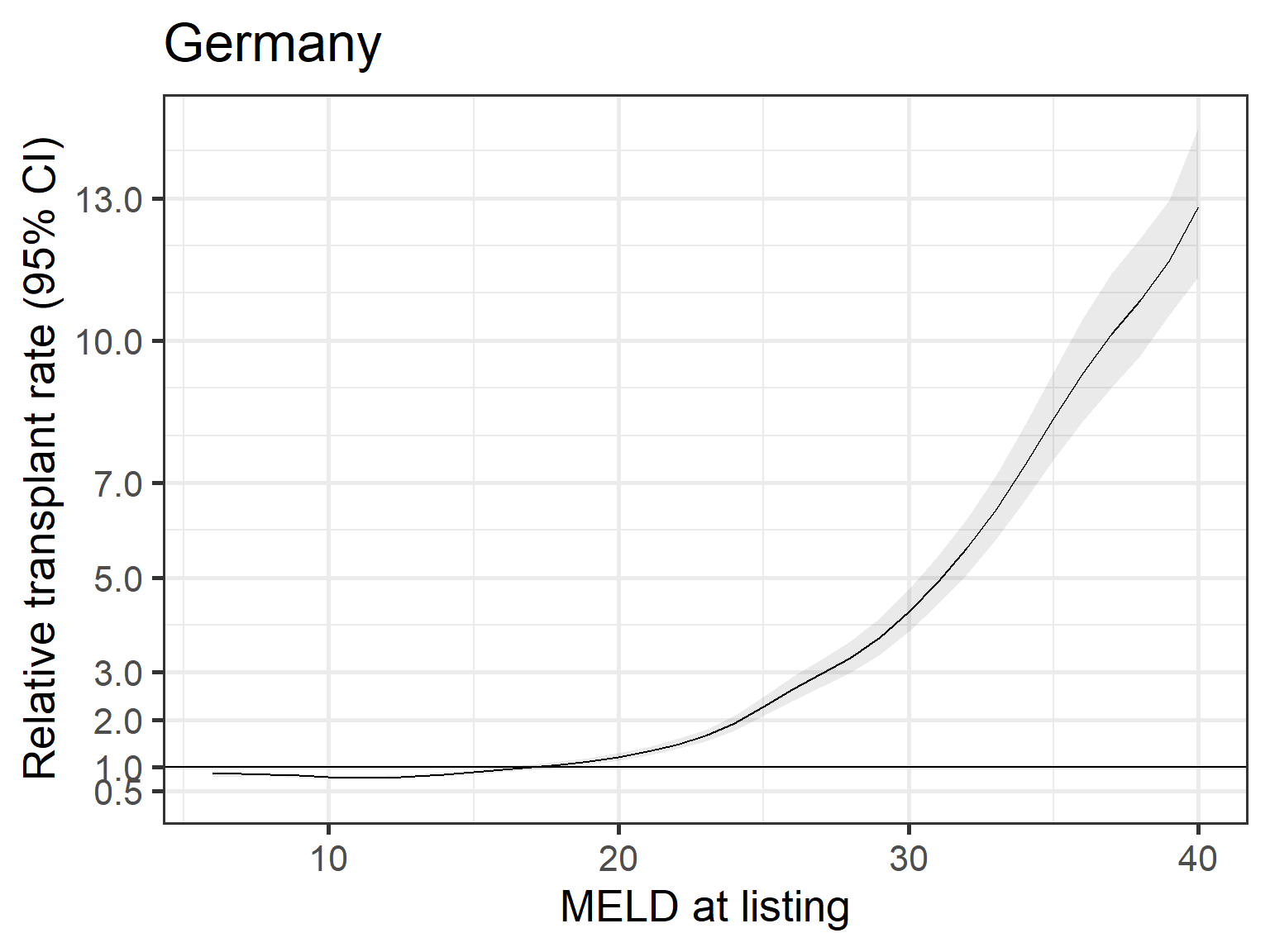
**Figure S8. Association of MELD score at listing with transplant rate.**

The chance of receiving an adequate organ offer in a MELD-based liver allocation system is depending on a high allocation MELD score. Therefore, the incorrectly missing MELD points of women have an actual negative impact on their transplantation rate. As shown, the chance of liver transplantation increases strongly with a higher MELD score at listing.

**References**

1. Kamath PS, Wiesner RH, Malinchoc M, et al. A model to predict survival in patients with end-stage liver disease. *Hepatology*. Feb 2001;33(2):464-70. doi:10.1053/jhep.2001.22172

2. Levey AS, Stevens LA, Schmid CH, et al. A new equation to estimate glomerular filtration rate. *Ann Intern Med*. May 5 2009;150(9):604-12. doi:10.7326/0003-4819-150-9-200905050-00006

3. R Core Team (2023). R: A Language and Environment for Statistical Computing. R Foundation for Statistical Computing. Vienna, Austria. <<https://www.R-project.org/>>

4. Wickham H, Averick M, Bryan J, et al. Welcome to the Tidyverse. *Journal of Open Source Software*. 2019;4(43)doi:10.21105/joss.01686

5. Gray B (2022). cmprsk: Subdistribution Analysis of Competing Risks. Version R package version 2.2-11. <https://CRAN.R-project.org/package=cmprsk>

6. Wickham H. ggplot2: Elegant Graphics for Data Analysis. Springer-Verlag New York; 2016.

7. Therneau T, Grambsch PM. Modeling Survival Data: Extending the Cox Model. Springer; 2000.

8. Therneau T, Lumley T, Atkinson E, Crowson C (2021). A Package for Survival Analysis in R. Version 3.2-13.
